# Molecular characteristics of KPC-producing *Klebsiella pneumoniae* isolates from children and adults in Kunming

**DOI:** 10.1128/spectrum.01813-26

**Published:** 2026-09-23

**Authors:** Wenting Wang, Jing Qian, Rui Zheng, Hanyu Liu, Xiaoli Jian, Jinglin Zhao, Yuanqing Liang, Lanhua Bao, Min Su, Yuan Chen, Yaofang Hang, Jianguo Wu, Xiao You, Adong Shen, Dingrui Han, Li Jiang, Xiaomei Liu

**Affiliations:** 1 Kunming Children’s Hospital (Kunming Medical University Affiliated), Kunming, China; 2 The First People’s Hospital of Kunming, Kunming, China; 3 The First People’s Hospital of Yunnan Province, Kunming, China; 4 Yunnan Provincial Hospital of Traditional Chinese Medicine, Kunming, China; Beijing Institute of Genomics, Chinese Academy of Sciences, Beijing, China

**Keywords:** Carbapenem-resistant *Klebsiella pneumoniae*, hypervirulence, pediatrics, ST11, whole-genome sequencing

## Abstract

The increasing prevalence of KPC-producing Klebsiella pneumoniae (KPC-Kp) poses a critical public health threat. While Sequence Type 11 (ST11) is the dominant carbapenem-resistant clone in China, the comparative molecular characteristics of isolates affecting distinct patient demographics remain understudied. Fifty non-duplicate KPC-Kp clinical isolates (18 from children, 32 from adults) were prospectively collected from four hospitals in Kunming during 2023. Whole-genome sequencing was performed to determine multilocus sequence types (MLST), capsular serotypes (KL types), plasmid replicons, and resistance/virulence gene profiles. Phylogenetic relationships were reconstructed using single-nucleotide polymorphisms and core-genome MLST. All isolates carried *bla_KPC-2_*, and 98% (49/50) belonged to ST11. Pediatric isolates were predominantly ST11-KL47 (72.2%), exhibiting a classic multidrug-resistant profile, whereas adult isolates were dominated by ST11-KL64 (59.4%) and frequently harbored IncFIB/IncH1IB virulence plasmids carrying rmpA, iuc, and iro genes, indicating a hypervirulent phenotype (CR-hvKP). Kunming isolates were genetically closely related to strains from other Chinese provinces, suggesting that local CRKP epidemiology is shaped by national dissemination of successful ST11 clones rather than isolated regional outbreaks. Although ST11 drives the regional KPC-Kp epidemic, the molecular characteristics of isolates from children and adults are distinct. The shared ST11 genetic backbone and the presence of transferable virulence plasmids in adult isolates raise a potential risk of cross-population spread. Continuous genomic surveillance is essential to monitor the possible horizontal transfer of virulence plasmids from adult to pediatric reservoirs.

**IMPORTANCE:**

This study investigated 50 KPC-2-producing Klebsiella pneumoniae isolates from four hospitals in Kunming (2023). Nearly all (98%) belonged to ST11, confirming this clone drives the regional carbapenem-resistant epidemic. However, striking differences emerged between pediatric and adult isolates. Pediatric isolates were predominantly ST11-KL47 (72.2%) with a classic multidrug-resistant profile, whereas adult isolates were mainly ST11-KL64 (59.4%) and frequently carried IncFIB/IncH1IB virulence plasmids harboring rmpA, iuc, and iro genes, indicating a hypervirulent phenotype (CR-hvKP). Phylogenetic analysis revealed that Kunming strains are closely related to those from other Chinese provinces, suggesting national dissemination of successful ST11 clones rather than isolated local outbreaks. Although adult virulence plasmids were not found in pediatric isolates, the shared ST11 genetic backbone raises concerns about potential horizontal transfer of these plasmids from adult to pediatric populations. Continuous genomic surveillance is essential to monitor this cross‑population risk.

## INTRODUCTION

*Klebsiella pneumoniae (K. pneumoniae) is* an opportunistic Gram-negative pathogen belonging to the family Enterobacteriaceae (1). Although it typically colonizes the upper respiratory and gastrointestinal tracts of humans, it is a leading cause of hospital-acquired (nosocomial) infections, including severe conditions such as pneumonia, urinary tract infections, surgical site infections, and life-threatening sepsis and meningitis. The global escalation of antimicrobial resistance (AMR) has critically undermined the efficacy of treatments for *K. pneumoniae* infections, contributing to increased morbidity, prolonged hospitalization, and higher mortality rates worldwide (2). The most significant mechanism driving this clinical crisis is the acquisition and expression of carbapenemases—enzymes that hydrolyze carbapenem antibiotics, which are often considered drugs of last resort for treating severe Gram-negative infections (3). This broad-spectrum β-lactam resistance has crystallized into a worldwide public health issue.

Carbapenemases are classified into three major Ambler classes based on their molecular mechanism. These include class A carbapenemases, primarily represented by Klebsiella pneumoniae carbapenemase (KPC); class B metallo-β-lactamases, such as New Delhi metallo-β-lactamase (NDM); and certain class D OXA-48-like carbapenemases (e.g., OXA-181, OXA-232, and OXA-163) (4). Among these, KPC, particularly the globally dominant variant KPC-2, stands out due to its high transmission potential and prevalence. The gene encoding KPC (bla_KPC_) is typically carried on mobile genetic elements, most notably plasmids, which facilitates the rapid horizontal transfer of resistance across different bacterial species and strains within the clinical environment (5). Furthermore, KPC-producing strains often belong to successful international high-risk clones, such as Sequence Type 258 (ST258) and its derivatives, which are recognized as international high-risk clones (i.e., lineages associated with global dissemination, multidrug resistance, and healthcare-associated outbreaks) (6). The emergence and spread of these Carbapenem-Resistant *K. pneumoniae* (CRKP) strains severely limit therapeutic options, often necessitating the use of highly toxic or less effective alternative antibiotics.

In China, the epidemiological landscape of CRKP has become increasingly alarming. Surveillance data from the China Antimicrobial Surveillance Network document a steady and concerning increase in the proportion of CRKP among clinical *K. pneumoniae* isolates, rising dramatically from 2.9% in 2005 to an average of 23.4% by 2024 (7). This pervasive increase underscores an urgent need for regional monitoring. Importantly, previous national and regional surveillance studies have highlighted distinct differences in the dominant carbapenemase types across patient demographics. KPC-producers have generally been found to be widespread in adult patients, whereas NDM-producers were frequently observed to be prevalent in pediatric cohorts, followed by KPC-2 and OXA-48-like producers in children (4). These findings suggest that the transmission dynamics and selective pressures operating in adult and pediatric healthcare environments may vary, resulting in different dominant resistance mechanisms. Understanding the precise molecular mechanisms and routes of cross-patient transmission is paramount to interrupting the spread of these pathogens.

Our previous investigations in Kunming have provided preliminary, yet crucial, insights into the CRKP situation. An initial study focusing on a major Kunming children’s hospital demonstrated high genetic diversity among CRKP isolates, suggesting potential transmission from multiple, unrelated sources (8). Following this, a broader multicenter study across a children's specialist hospital and three general hospitals confirmed that KPC was the most prevalent carbapenemase type across the combined cohort, dominating the regional CRKP landscape. Critically, this multicenter analysis, utilizing Repetitive Element Palindromic (REP)-PCR genotyping, indicated that some isolates from adult and child patients clustered into the same clonal types (9). While REP-PCR suggested potential cross-regional and cross-demographic spread, its discriminatory power is inherently limited. To accurately map the transmission pathways, confirm the clonal identity at high resolution, and identify the mobile genetic elements responsible for resistance dissemination between adult and pediatric populations, the use of Whole-Genome Sequencing (WGS) is essential. WGS provides nucleotide-level resolution to distinguish between closely related isolates (i.e., true clonal transmission) and unrelated acquisition events. Therefore, this study aims to leverage WGS to precisely determine the molecular characteristics and clonal dissemination patterns of KPC-producing *K. pneumoniae* isolates collected from children and adults within Kunming. Specifically, we will characterize the sequence types (STs), identify associated resistance and virulence genes, and define the genetic basis (e.g., specific plasmids) mediating the observed clonal relatedness between adult and pediatric isolates, thereby providing critical evidence to inform regional infection control policies.

## MATERIALS AND METHODS

### Bacterial collection

A total of 50 non-duplicate KPC-producing Klebsiella pneumoniae (KPC-Kp) isolates were prospectively collected in Kunming City throughout 2023. The collection was stratified by patient cohort: 18 pediatric isolates were obtained from a dedicated children specialist hospital, and 32 adult isolates were collected from 3 distinct general hospitals (Hospital A: 15 isolates; Hospital B: 13 isolates; Hospital C: 4 isolates).

### Phenotypic characterization and antimicrobial susceptibility testing

Species identification was confirmed using the automated VITEK-2 Compact system (BioMérieux, Marcy l’Etoile, France). Antimicrobial susceptibility testing (AST) for imipenem was determined via the standard Kirby-Bauer disk diffusion method, with results interpreted according to the Clinical and Laboratory Standards Institute guidelines (10). Escherichia coli ATCC 25922 was utilized as the quality control strain for AST. Carbapenemase production was phenotypically confirmed using a commercial disk diffusion kit (DL Biotech, Zhuhai, China) in accordance with the Chinese expert consensus on CRKP detection (11). Imipenem (IPM) disks were supplemented with three inhibitors: ethylenediamine tetraacetic acid (EDTA), 3-aminophenylboronic acid hydrochloride (APB), and a combination of EDTA plus APB. The presence of a class A carbapenemase (KPC) was concluded if the inhibition zone diameter around the IPM disc combined with APB (or APB plus EDTA) increased by >= 5 mm compared to the plain IPM disk control.

### Whole-genome sequencing and assembly

Genomic DNA was extracted from each isolate using the HiPure Bacterial DNA Kit (Magen, Guangzhou, China). Libraries for second-generation sequencing were prepared using the pathogen library construction kit (Genskey, Tianjin, China), which involved enzymatic fragmentation, end repair, adaptor ligation, purification, and final amplification. Sequencing was performed on the Genetron S2000 platform (Genetron Health, Beijing, China). Raw sequencing reads underwent quality trimming and *de novo* assembly utilizing the Shovill pipeline (version 1.0.1) (https://github.com/tseemann/shovill). Assembly quality was verified by visualizing the draft genomes using the BLAST Ring Image Generator (BRIG) software (https://sourceforge.net/projects/brig/) aligned against a reference *K. pneumoniae* genome (GenBank accession number CP003200) (12) and local Kunming plasmids (13) (GenBank accession numbers CP053017, CP053018, CP053019, CP053020).

### Genomic and comparative phylogenetic analysis

Bioinformatic analyses were performed on the assembled genomes. Genomes were initially annotated using Prokka 1.11.

#### Sequence and Plasmid Typing

Multi-Locus Sequence Typing (MLST) and capsule K typing were determined by analyzing the allelic profiles of seven housekeeping genes (gapA, infB, mdh, pgi, phoE, rpoB, and tonB) against the *K. pneumoniae* MLST scheme in the Bacterial Isolate Genome Sequence Database (BIGSDB, https://bigsdb.pasteur.fr/). Plasmid replicon types were identified using the PlasmidFinder 2.1.6 tool (https://cge.food.dtu.dk/services/PlasmidFinder/), requiring an alignment identity score of >= 90%.

#### Resistance and Virulence Profiling

Antibiotic resistance genes were predicted by comparison against the Comprehensive Antibiotic Resistance Database (https://card.mcmaster.ca/). Virulence factors were identified via BLASTP 2.5.0+ searches against the Virulence Factor Database (http://www.mgc.ac.cn/VFs/). The hypervirulent status of strains was analyzed using the K-PAM platform (https://www.iith.ac.in/K-PAM/).

#### Local and National Phylogeny

A maximum-likelihood (ML) phylogenetic tree for the 50 Kunming isolates was constructed based on Single-Nucleotide Polymorphisms (SNPs) identified in the core genome using KSNP4 (k-mer = 19). The tree was visualized and annotated using Chiplot online software (https://www.chiplot.online/). To establish a national context, core-genome Multi-Locus Sequence Typing (cgMLST) analysis was performed. The Kunming isolates were compared against a national panel of 61 publicly available class A carbapenemase-producing strains from other regions (including 58 Sequence Type 11 [ST11] and 3 ST1326 strains), using *K. pneumoniae* HS11286 as the reference strain. The resulting cgMLST tree was constructed via the BacWGSTdb 2.0 web platform (http://www.bacdb.cn/BacWGSTdb/).

## RESULTS

### Clinical characteristics of the KPC-producing CRKP isolates

A total of 50 KPC-producing *K. pneumoniae* strains were analyzed in this study. The collection comprised 18 isolates (36.0%) from a pediatric specialist hospital and 32 isolates (64.0%) from 3 general adult hospitals in Kunming. The clinical sources of these isolates were diverse, reflecting the opportunistic nature of the pathogen. As detailed in Table S1, most isolates were recovered from respiratory tract specimens, specifically sputum (*n* = 35, 70%), followed by other specimen types including hydrothorax, secretion, midstream urine, blood, bile, and cerebrospinal fluid.

### Assembly of chromosomes and plasmids

**Fig 1:**
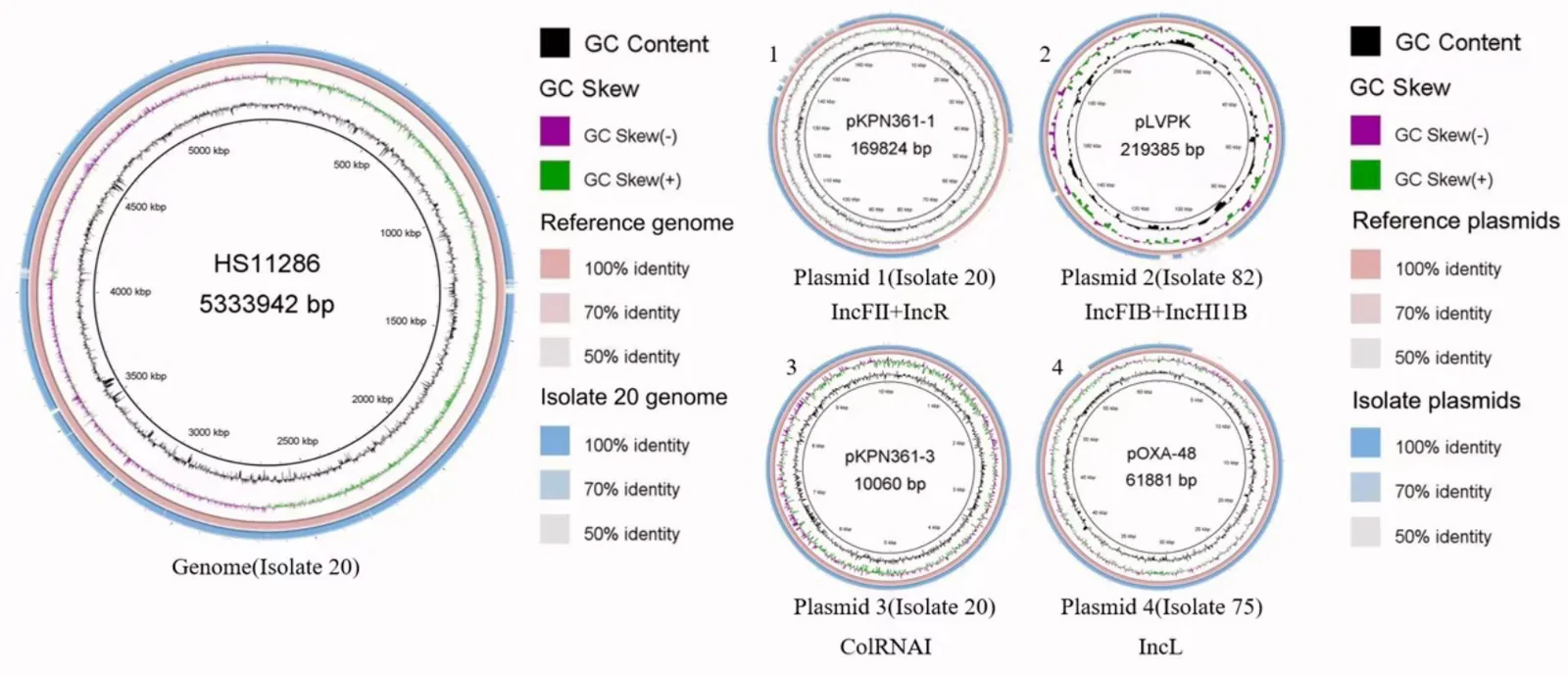
Circular representation of the genome including the chromosome and four plasmids. The center of the circle diagram indicated the name and size of the reference core genome. The structures of the diagram from inside to outside are as follows: GC content, GC sketch, complete structure of the core genome of strain HS11286, and core genome structure of isolate 9.

Whole-genome sequencing and assembly were performed to characterize the genetic architecture of the isolates. The genomic structure of a representative strain (Isolate 20) exhibited high similarity to the reference genome *K. pneumoniae* HS11286, as illustrated in the circular genome map (Fig. 1).

Plasmid replicon analysis revealed a high prevalence of specific plasmid types among the studied cohort (Table 1). The ColRNAI and IncFII (pHN7A8) plasmids were ubiquitous, detected in 100% and 96% (48/50) of isolates, respectively. Notably, the distribution of other plasmids varied significantly by patient population. The IncR plasmid was highly prevalent in both groups but slightly higher in adults (96.9% vs 83.3%). Conversely, the IncFIB (K) and IncHI1B (pNDM-MAR) plasmids showed a marked predilection for the adult cohort. IncFIB (K) was detected in 59.4% of adult isolates compared to only 11.1% of pediatric isolates, and IncHI1B was found in 53.1% of adult isolates vs 11.1% in children.

**TABLE 1:** Frequency of plasmid replicon types detected across the 50 CRKP isolates, comparing prevalence between the pediatric and adult cohorts

Plasmid type	Children specialist hospital *n* = 18		Comprehensive hospital *n* = 32		Fisher’s exact test *P*-value
	Number	Proportion %	Number	Proportion %	
ColRNAI	18	100.0	32	100.0	1
IncFII (pHN7A8)	18	100.0	30	93.8	0.530
IncR	15	83.3	31	96.9	0.127
IncFIB (K)	2	11.1	19	59.4	0.001
IncHI1B (pNDM-MAR)	2	11.1	17	53.1	0.005
IncL	0	0.0	1	3.1	1

Analysis of plasmid co-carriage patterns (Table 2) further highlighted these demographic distinctions. The combination of “ColRNAI + IncFII + IncR” was the dominant pattern in the pediatric group (61.1%), whereas the more complex “ColRNAI + IncFII + IncR + IncFIB + IncHI1B” pattern was significantly more frequent in the adult group (46.9% vs 16.7%; *P* = 0.05). Additionally, one adult isolate carried an IncL plasmid, typically associated with *bla*_OXA-48_.

**TABLE 2:** Comparison of plasmid co-carriage patterns between pediatric and adult CRKP isolates, highlighting statistical differences in plasmid complexity

Plasmid carrying pattern	Children specialist hospital *n* = 18		Comprehensive hospital *n* = 32		Fisher’s exact test *P*-value
	Number	Proportion %	Number	Proportion %	
ColRNAI + IncFII + IncR	11	61.1	13	40.6	0.05
ColRNAI + IncFII + IncR + IncFIB + IncHI1B	3	16.7	15	46.9	
ColRNAI + IncFII	3	16.7	1	3.1	
ColRNAI + IncFII + IncR + IncFIB ColRNAI + IncFII + IncR + IncL ColRNAI + IncR + IncFIB + IncHI1B	0	0.0	1	3.1	
	0	0.0	1	3.1	
	0	0.0	1	3.1	
ColRNAI + IncR + IncFIB	1	5.6	0	0.0	

### Sequence typing and capsular serotypes

Multi-Locus Sequence Typing identified that the vast majority of the isolates (98%, 49/50) belonged to Sequence Type 11 (ST11), the predominant CRKP clone in China. Only a single isolate from the pediatric group was identified as ST1326 (Table 3).

**TABLE 3:** Distribution of multi-locus sequence types (MLST) and capsular serotypes (KL-types) among the 50 KPC-producing *K. pneumoniae* isolates, stratified by patient group (children vs adults)

MLST typing	Capsular serotypes	Children specialist hospital *n* = 18		Comprehensive hospital *n* = 32		Fisher’s exact test *P*-value
		Number	Proportion %	Number	Proportion %	
ST11	KL47	13	72.2	12	37.5	＜0.01
	KL64	3	16.6	19	59.4	
	KL25	1	5.6	1	3.1	
ST1326	KL110	1	5.6	-	-	

**Fig 2:**
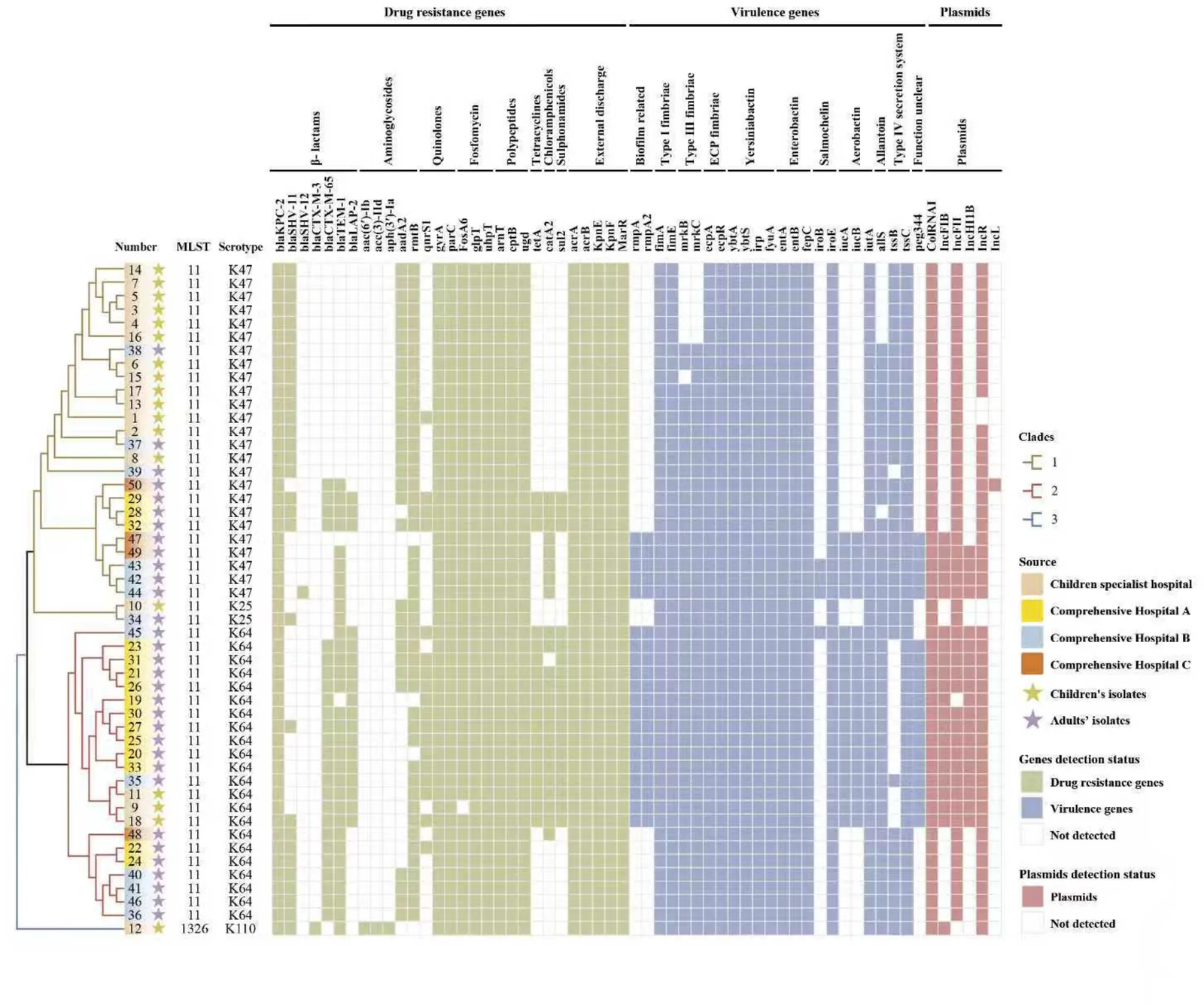
The phylogenetic tree and the distribution of antibiotic resistance genes of the 50 CRKP strains. SNP phylogenetic tree, MLST sequence types, capsular serotypes and heatmap of plasmids, resistance and virulence genes to the 50 CRKP isolates. The text annotations above the heatmap from top to bottom are gene classification, the function of genes in resistance or virulence, and type of genes or plasmids. The isolates’ clade, source hospital, age group, and whether corresponding plasmids, virulence, or resistance genes have been detected of the CRKP are all marked with different colors.

Despite the dominance of ST11, there was a statistically significant divergence in capsular serotypes (KL-types) between adults and children (Table 3; Fig. 2). The pediatric cohort was dominated by the KL47 serotype (72.2%, 13/18), whereas the adult cohort was predominantly characterized by the KL64 serotype (59.4%, 19/32). This distinct lineage segregation suggests that while ST11 is the primary driver of the epidemic in Kunming, different sub-lineages are circulating within pediatric and adult populations (*P* < 0.01).

*Distribution and characteristics of drug resistance and virulence genes*: The genomic landscape of antimicrobial resistance and virulence was visualized via a heatmap (Fig. 2). All isolates harbored the bla*_KPC-2_* gene, consistent with the inclusion criteria. The resistance profile was extensive, with a high prevalence of non-β-lactam resistance genes, including those conferring resistance to aminoglycosides (*rmtB*), quinolones (*qnrS1*), and sulfonamides (*sul2*).

Virulence gene profiling revealed distinct clustering associated with the capsular types described above. The SNP-based phylogenetic tree (Fig. 2) segregated the isolates into three main clades. Clades 1 and 2 were largely composed of KL47 isolates (predominantly children) and exhibited a baseline virulence profile typified by the presence of yersiniabactin (*ybt*, *irp*, *fyuA*) and biofilm-related genes (*mrkD*). In contrast, Clade 3 was dominated by KL64 isolates (predominantly adults) and displayed a hypervirulent genetic signature. These isolates frequently co-harbored genes associated with salmochelin (*iro*), aerobactin (*iuc*), and the hypermucoviscosity regulators (*rmpA/rmpA2*). This suggests that the ST11-KL64 sub-lineage circulating in adults has acquired a virulence plasmid, evolving into a carbapenem-resistant hypervirulent *K. pneumoniae* (CR-hvKP) phenotype.

### Phylogenetic network

**Fig 3:**
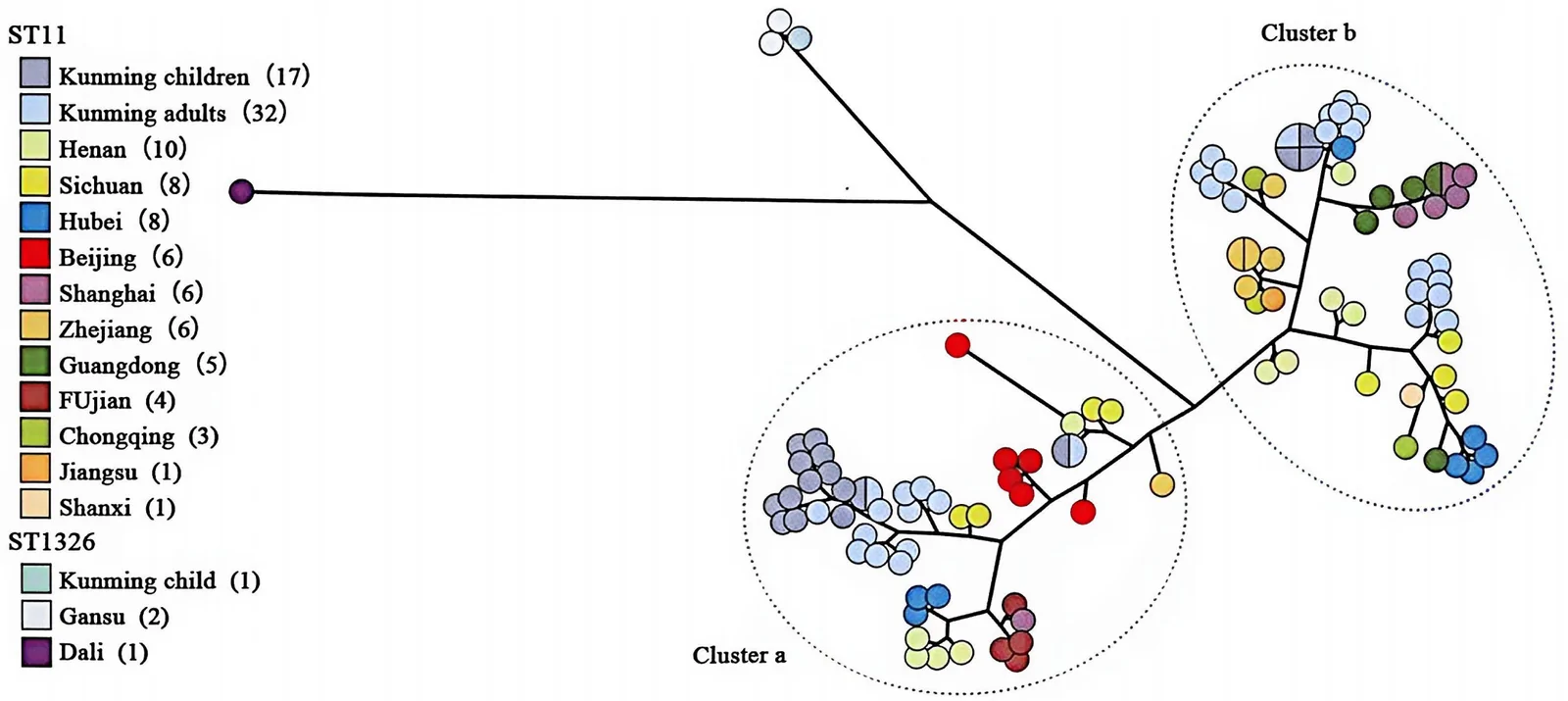
The cgMLST phylogenetic network of 107 ST11 CRKP isolates and 4 ST1326 isolates. The color of the circle represents a specific region, and the line length between each circle denotes the genetic relationships of different isolates. The numbers in brackets indicate the quantity of isolates from that region.

To contextualize the Kunming isolates within the national epidemiology of CRKP, a cgMLST analysis was conducted including 111 strains (50 from this study and 61 publicly available genomes from across China, detailed in Table S2). The resulting minimum spanning tree (Fig. 3) revealed that the ST11 isolates formed two major clusters (Cluster a and Cluster b).

The Kunming isolates (represented in purple for children and light blue for adults) did not form a single monophyletic cluster exclusive to the region. Instead, they were distributed throughout the network, showing close genetic relatedness to strains from geographically distant provinces such as Henan, Sichuan, and Zhejiang. This phylogenetic interleaving indicates that the CRKP strains circulating in Kunming were not isolated local variants but were part of the broader dissemination of successful ST11 sub-lineages across China.

## DISCUSSION

This study provided a high-resolution genomic characterization of KPC-producing *Klebsiella pneumoniae* isolates circulating in Kunming, revealing a striking molecular divergence between pediatric and adult populations despite a shared geographic locale. Our primary finding confirmed that ST11 remains the absolute dominant clone (98%) driving the regional CRKP epidemic, consistent with national surveillance data. However, whole-genome sequencing uncovered a significant lineage segregation: pediatric infections were predominantly caused by the KL47 serotype with a “classic” multidrug-resistant profile, whereas adult infections were dominated by the KL64 serotype exhibiting a hypervirulent (CR-hvKP) phenotype. Furthermore, we identified that this hypervirulence in adults is inextricably linked to the acquisition of specific IncFIB and IncHI1B plasmids carrying key virulence determinants (*rmpA*, *iuc*, *iro*). These results suggested that while ST11 was the common vehicle for carbapenem resistance in Kunming, distinct evolutionary pressures or transmission networks were shaping the sub-lineages affecting children vs adults.

The dominance of ST11-KPC-2 observed in our cohort mirrors the established epidemiological baseline in China, where ST11 is recognized as the primary high-risk clone responsible for the dissemination of carbapenem resistance. However, the capsular serotype distribution adds a critical layer of complexity. Previous studies have proposed an evolutionary model for ST11, suggesting that the ST11-KL64 lineage emerged from an ancestral ST11-KL47 clone through capsular switching and the acquisition of virulence plasmids (14, 15). Our data strongly supports this hypothesis but adds a demographic dimension: the adult population in our cohort appears to be the predominant source of this putative hypervirulent sub-lineage, but population-based carriage studies are needed to confirm this reservoir status. In contrast, the pediatric population retains the ST11-KL47 lineage. This discrepancy might reflect different antibiotic pressures, hospital transmission dynamics, or a lag in the introduction of the hypervirulent clone into pediatric wards. The persistence of KL47 in children is noteworthy and aligns with findings from other pediatric cohorts where non-hypervirulent CRKP strains remain prevalent (16, 17), contrasting with the rapid replacement of KL47 by KL64 reported in adult intensive care units across China.

A pivotal finding of this study is the correlation between plasmid content and the hypervirulent phenotype. The adult isolates frequently harbored a complex plasmid array including IncFIB and IncHI1B, which are known vectors for hypervirulence genes (18). The co-occurrence of *bla*_KPC-2_ with *rmpA/rmpA2* and siderophore gene clusters (*iuc*, *iro*) in the adult KL64 cohort confirms the establishment of bona fide CR-hvKP in Kunming. This convergence of high virulence and high resistance poses a catastrophic clinical threat, as these strains are capable of causing severe, metastatic infections in immunocompetent hosts while remaining resistant to last-line antibiotics (19). Conversely, the pediatric isolates were characterized by a simpler plasmid backbone (ColRNAI + IncFII + IncR) and generally lacked these hypervirulence-associated elements. While this suggests that children in this region are currently spared from the CR-hvKP superbug, the presence of identical core ST11 genetics implies that the potential for plasmid transfer exists (20). Continuous surveillance is required to detect any shift of these virulence plasmids into the pediatric bacterial reservoir.

Phylogenetically, the Kunming isolates did not form a monophyletic “local” cluster but were interspersed with strains from geographically diverse provinces such as Henan, Sichuan, and Zhejiang. This suggests that the CRKP burden in Kunming is not the result of a single, isolated outbreak but rather continuous importation and dissemination of successful national clones. The high connectivity of the ST11 lineage across China highlights the ease with which these pathogens travel across regional borders, likely facilitated by patient transfer and the high mobility of the population (21).

The primary strength of this study is the use of WGS to compare paired pediatric and adult cohorts within the same city, offering a comparative snapshot that standard surveillance often overlooks. This approach allowed us to identify the sub-lineage segregation (KL47 vs KL64) that traditional phenotypic methods would have missed. However, there are limitations. First, the sample size (*n* = 50) is relatively small, which may limit the generalizability of the statistical associations. Second, the study is limited to a single year (2023) and a specific city, so it may not fully capture longitudinal evolutionary trends. Third, detailed clinical information and patient outcomes were not available for analysis, which limits our ability to correlate specific genotypes or virulence genes with the actual pathogenic potential of the isolates. Finally, while we identified virulence genes, *in vivo* virulence assays (e.g., *Galleria mellonella* infection models) were not performed to phenotypically confirm hypervirulence although the genetic signatures identified are widely accepted predictors of the hypervirulent phenotype.

### Conclusion

While the ST11 clone is the predominant driver of KPC-producing *K. pneumoniae* infections in Kunming, the molecular characteristics of isolates from children and adults are distinct. The adult population is characterized by the emergence of the putative hypervirulent ST11-KL64 sub-lineage carrying virulence plasmids, whereas children are predominantly affected by the classic ST11-KL47 MDR lineage. Kunming isolates are genetically closely related to strains from other Chinese provinces. This indicates that the local CRKP epidemiology is shaped by the national dissemination of successful ST11 clones rather than by isolated regional outbreaks. Importantly, while pediatric isolates in this study exhibited a less virulent profile, the shared ST11 genetic backbone and the presence of transferable virulence plasmids in adult isolates raise a potential risk of cross-population spread. However, our data did not permit definitive inferences regarding transmission dynamics, and larger-scale studies incorporating carriage surveillance are warranted to clarify these risks. Therefore, continuous genomic surveillance is essential to monitor the possible horizontal transfer of virulence plasmids from adult to pediatric reservoirs.

## Data Availability

The clean sequence data supporting the findings of this study have been deposited in the Genome Sequence Archive (GSA) at the National Genomics Data Center (NGDC) under the project number CRA026279. The data are publicly accessible.

## SUPPLEMENTAL MATERIAL

Tables S1 and S2 spectrum.01813-26-s0001.docx Table S1: Clinical characteristics of 18 children's CRKP isolates and 32 adults' isolates at four hospitals in Kunming City, China, 2023. Table S2: Genomic information of 58 ST11 CRKP isolates and 3 ST1326 CRKP isolates from different regions of China.

ASM does not own the copyrights to Supplemental Material that may be linked to, or accessed through, an article. The authors have granted ASM a non-exclusive, world-wide license to publish the Supplemental Material files. Please contact the corresponding author directly for reuse.
