# Supplementary material for: Molecular characteristics of KPC-producing Klebsiella pneumoniae isolates from children and adults in Kunming
Source: Microbiol Spectr. 2026 Sep 23;14(11):e01813-26. doi: 10.1128/spectrum.01813-26 (PMC13729193; doi:10.1128/spectrum.01813-26)
Supplement: Supplementary file 1 [file spectrum.01813-26-s0001.docx]

**Supplemental Table 1.** Clinical characteristics of 18 children's CRKP isolates and 32 adults' isolates at four hospitals in Kunming City, China, 2023.

| ID | Hospital | patient | Clinical Sample |
| --- | --- | --- | --- |
| 1 | Children specialist hospital | child | sputum |
| 2 | Children specialist hospital | child | anal swab |
| 3 | Children specialist hospital | child | sputum |
| 4 | Children specialist hospital | child | sputum |
| 5 | Children specialist hospital | child | sputum |
| 6 | Children specialist hospital | child | sputum |
| 7 | Children specialist hospital | child | sputum |
| 8 | Children specialist hospital | child | sputum |
| 9 | Children specialist hospital | child | sputum |
| 10 | Children specialist hospital | child | secretion |
| 11 | Children specialist hospital | child | sputum |
| 12 | Children specialist hospital | child | sputum |
| 13 | Children specialist hospital | child | midstream urine |
| 14 | Children specialist hospital | child | sputum |
| 15 | Children specialist hospital | child | sputum |
| 16 | Children specialist hospital | child | sputum |
| 17 | Children specialist hospital | child | sputum |
| 18 | Children specialist hospital | child | sputum |
| 19 | Comprehensive Hospital A | adult | sputum |
| 20 | Comprehensive Hospital A | adult | hydrothorax |
| 21 | Comprehensive Hospital A | adult | sputum |
| 22 | Comprehensive Hospital A | adult | sputum |
| 23 | Comprehensive Hospital A | adult | sputum |
| 24 | Comprehensive Hospital A | adult | sputum |
| 25 | Comprehensive Hospital A | adult | midstream urine |
| 26 | Comprehensive Hospital A | adult | sputum |
| 27 | Comprehensive Hospital A | adult | blood |
| 28 | Comprehensive Hospital A | adult | sputum |
| 29 | Comprehensive Hospital A | adult | bile |
| 30 | Comprehensive Hospital A | adult | hydrothorax |
| 31 | Comprehensive Hospital A | adult | cerebrospinal fluid |
| 32 | Comprehensive Hospital A | adult | hydroperitoneum |
| 33 | Comprehensive Hospital A | adult | hydroperitoneum |
| 34 | Comprehensive Hospital B | adult | sputum |
| 35 | Comprehensive Hospital B | adult | sputum |
| 36 | Comprehensive Hospital B | adult | sputum |
| 37 | Comprehensive Hospital B | adult | sputum |
| 38 | Comprehensive Hospital B | adult | sputum |
| 39 | Comprehensive Hospital B | adult | sputum |
| 40 | Comprehensive Hospital B | adult | sputum |
| 41 | Comprehensive Hospital B | adult | sputum |
| 42 | Comprehensive Hospital B | adult | sputum |
| 43 | Comprehensive Hospital B | adult | sputum |
| 44 | Comprehensive Hospital B | adult | sputum |
| 45 | Comprehensive Hospital B | adult | sputum |
| 46 | Comprehensive Hospital B | adult | sputum |
| 47 | Comprehensive Hospital C | adult | midstream urine |
| 48 | Comprehensive Hospital C | adult | secretion |
| 49 | Comprehensive Hospital C | adult | sputum |
| 50 | Comprehensive Hospital C | adult | sputum |

**Supplemental Table 2.** Genomic information of 58 ST11 CRKP isolates and 3 ST1326 CRKP isolates from different regions of China.

| Number | BioSample ID | GCA Number | Download  Website | MLST Type | Region |
| --- | --- | --- | --- | --- | --- |
| 1 | SAMN18087493 | GCA_017291515.1 | NCBI | ST11 | Henan |
| 2 | SAMN18087495 | GCA_017291825.1 | NCBI | ST11 | Henan |
| 3 | SAMN18087498 | GCA_017291835.1 | NCBI | ST11 | Henan |
| 4 | SAMN18087501 | GCA_017292055.1 | NCBI | ST11 | Henan |
| 5 | SAMN18087514 | GCA_017291635.1 | NCBI | ST11 | Henan |
| 6 | SAMN30359372 | GCA_031800775.1 | NCBI | ST11 | Henan |
| 7 | SAMN30359458 | GCA_031800755.1 | NCBI | ST11 | Henan |
| 8 | SAMN30359369 | GCA_031800735.1 | NCBI | ST11 | Henan |
| 9 | SAMN30359367 | GCA_031800815.1 | NCBI | ST11 | Henan |
| 10 | SAMN30359348 | GCA_031801115.1 | NCBI | ST11 | Henan |
| 11 | [SAMN28546263](https://www.ncbi.nlm.nih.gov/biosample/SAMN28546263) | GCA_023572965.1 | NCBI | ST11 | Sichuan |
| 12 | SAMN28546299 | GCA_023890885.1 | NCBI | ST11 | Sichuan |
| 13 | SAMN28546270 | GCA_023893355.1 | NCBI | ST11 | Sichuan |
| 14 | SAMN28546389 | GCA_023887885.1 | NCBI | ST11 | Sichuan |
| 15 | SAMN14379834 | GCA_014655995.1 | NCBI | ST11 | Sichuan |
| 16 | SAMN14379836 | GCA_014655935.1 | NCBI | ST11 | Sichuan |
| 17 | SAMN14379843 | GCA_014655755.1 | NCBI | ST11 | Sichuan |
| 18 | SAMN14379847 | GCA_014655675.1 | NCBI | ST11 | Sichuan |
| 19 | SAMN27923381 | GCA_024436675.1 | NCBI | ST11 | Hubei |
| 20 | SAMN27923420 | GCA_024436575.1 | NCBI | ST11 | Hubei |
| 21 | SAMN27923436 | GCA_024436635.1 | NCBI | ST11 | Hubei |
| 22 | SAMN31207069 | GCA_028219955.1 | NCBI | ST11 | Hubei |
| 23 | SAMN31207059 | GCA_028221005.1 | NCBI | ST11 | Hubei |
| 24 | SAMN31207042 | GCA_028221855.1 | NCBI | ST11 | Hubei |
| 25 | SAMN31207039 | GCA_028224675.1 | NCBI | ST11 | Hubei |
| 26 | SAMN31207030 | GCA_028226565.1 | NCBI | ST11 | Hubei |
| 27 | SAMN33870550 | GCA_029455295.1 | NCBI | ST11 | Zhejiang |
| 28 | [SAMN16205033](https://www.ncbi.nlm.nih.gov/biosample/SAMN16205033) | GCA_025583855.1 | NCBI | ST11 | Zhejiang |
| 29 | SAMN16205035 | GCA_025583835.1 | NCBI | ST11 | Zhejiang |
| 30 | SAMN16205038 | GCA_025583775.1 | NCBI | ST11 | Zhejiang |
| 31 | SAMN30637424 | GCA_029291275.1 | NCBI | ST11 | Zhejiang |
| 32 | SAMN30637431 | GCA_029291125.1 | NCBI | ST11 | Zhejiang |
| 33 | [SAMN27593772](https://www.ncbi.nlm.nih.gov/biosample/SAMN27593772) | GCA_023700655.1 | NCBI | ST11 | Beijing |
| 34 | SAMN27593775 | GCA_023700645.1 | NCBI | ST11 | Beijing |
| 35 | SAMN04532756 | GCA_001613715.1 | NCBI | ST11 | Beijing |
| 36 | SAMN04534543 | GCA_001613665.1 | NCBI | ST11 | Beijing |
| 37 | SAMN04534541 | GCA_001613655.1 | NCBI | ST11 | Beijing |
| 38 | SAMN04534542 | GCA_001613645.1 | NCBI | ST11 | Beijing |
| 39 | SAMN28171616 | GCA_025572605.1 | NCBI | ST11 | Shanghai |
| 40 | SAMN28171615 | GCA_025572545.1 | NCBI | ST11 | Shanghai |
| 41 | SAMN28171622 | GCA_025572445.1 | NCBI | ST11 | Shanghai |
| 42 | SAMN28171624 | GCA_025572385.1 | NCBI | ST11 | Shanghai |
| 43 | SAMN28171620 | GCA_025572465.1 | NCBI | ST11 | Shanghai |
| 44 | SAMN06109054 | GCA_001902475.1 | NCBI | ST11 | Shanghai |
| 45 | SAMN31632941 | GCA_026154245.1 | NCBI | ST11 | Guangdong |
| 46 | SAMN31632931 | GCA_026154105.1 | NCBI | ST11 | Guangdong |
| 47 | SAMN31632946 | GCA_026154085.1 | NCBI | ST11 | Guangdong |
| 48 | SAMN31632938 | GCA_026154065.1 | NCBI | ST11 | Guangdong |
| 49 | SAMN31632943 | GCA_026154045.1 | NCBI | ST11 | Guangdong |
| 50 | SAMN10473945 | - | Pathogenwatch | ST11 | Fujian |
| 51 | SAMN10473949 | - | Pathogenwatch | ST11 | Fujian |
| 52 | SAMN10473950 | - | Pathogenwatch | ST11 | Fujian |
| 53 | SAMN10473951 | - | Pathogenwatch | ST11 | Fujian |
| 54 | SAMN19736494 | GCA_019134695.1 | NCBI | ST11 | Chongqing |
| 55 | SAMN30403289 | GCA_024762135.1 | NCBI | ST11 | Chongqing |
| 56 | SAMN31461329 | - | Pathogenwatch | ST11 | Chongqing |
| 57 | SAMN30234221 | GCA_027980205.1 | NCBI | ST11 | Shanxi |
| 58 | SAMN32767760 | GCA_028831855.1 | NCBI | ST11 | Jiangsu |
| 59 | SAMN19015924 | - | Pathogenwatch | ST1326 | Gansu |
| 60 | SAMN19015934 | - | Pathogenwatch | ST1326 | Gansu |
| 61 | SAMN20294500 | GCA_022592575.1 | NCBI | ST1326 | Dali |
